# Shedding light on arctic-alpine plants: the understudied photobiological responses in the context of climate change

**DOI:** 10.3389/fpls.2026.1920913

**Published:** 2026-08-07

**Authors:** Anna Cazzavillan, Thomas Abeli, Lisa Brancaleoni, Maurizio Cutini, Martina Tarascio, Renato Gerdol

**Affiliations:** 1 Department of Environmental and Prevention Sciences, University of Ferrara, Ferrara, Italy; 2 Department of Earth and Environmental Sciences, University of Pavia, Pavia, Italy; 3 Department of Science, ROMA TRE University, Rome, Italy

**Keywords:** arctic-alpine plants, climate change, photoperiod, spectral light, tundra

## Abstract

Arctic-alpine plant populations can be found across latitudinal, altitudinal, micro-topographical and abiotic gradients within the tundra biome, thus experiencing unique combinations of microclimatic niches, geological substrates and light environments. Rising temperatures have been the focus in arctic-alpine research, yet the interest in photic barriers and spectral light requirements is rising in current research. Although ecotypic differences across latitudes and altitudes have been observed and circadian activity under continuous light has been investigated, arctic-alpine photobiology is critically understudied despite its eco-physiological relevance. Experimental studies considering light among the abiotic factors, either for its relevance for photosynthesis or as a potential cue regulating phenology, are not only scarce but they also showcase marked heterogeneity in terms of experimental designs, with only few studies offering comparisons among populations. We argue that arctic-alpine plant species are fitting photobiological research targets thanks to their realized niches and ecological relevance.

## 1 Introduction: warming is not enough to study climate change responses

The rising interest and concern on global warming during the late 20th century, with the Villach Conference in 1985, the IPCC establishment in 1988 and the first IPCC report in 1990, formed the scientific substratum for the first International Tundra Experiment (ITEX) in December 1990 (Webber and Walker, 1991). The resulting protocol established a network of arctic and alpine study sites characterized by low-cost and low-impact manipulations of near-surface air temperature and snowmelt regimes (Molau and Mølgaard, 1996). Subsequently, a number of studies showed that light is a crucial yet understudied environmental factor when it comes to plant responses under climate change (Saikkonen et al., 2012; Tomiolo and Ward, 2018; Tarascio et al., 2026) but also predicted photic constraints to poleward range-shifts of plant species (Huffeldt, 2020).

**Figure 1:**
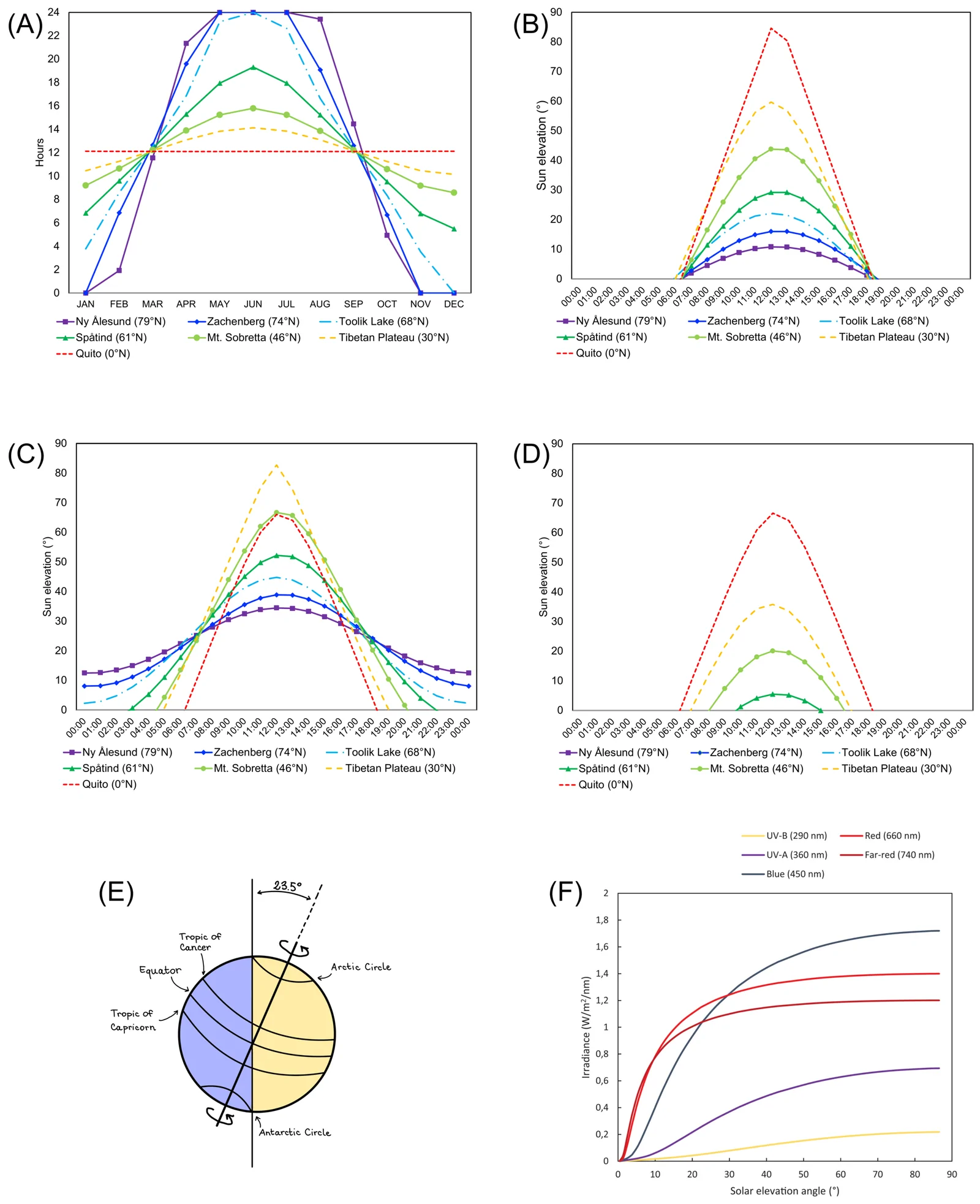
Light environment along a latitudinal gradient. **(A)** Mean monthly hours of daylight across a latitudinal gradient. Sun elevation angle (°) is assessed **(B)** for the March equinox, **(C)** the June solstice, and **(D)** the December solstice. **(E)** Drawing of the Earth during the June solstice. **(F)** Irradiance (SPCTRAL2[Bir86]) at different solar elevations relative to the horizon for UV‐A, UV‐B, blue, red, and far‐red light at 290, 360, 450, 660, and 740 nm, respectively, from Mølmann et al., 2021, Physiologia Plantarum, Volume: 172, Issue: 4, Pages: 1931–1940, First published: 10 April 2021, DOI: (10.1111/ppl.13418). See Appendix 1: **Supplementary Tables S1**–**S4**, for supporting data used in the graphs **(A–D)**.

Photoperiod is currently considered as one of the major factors impacting range shifts of plant species in response to the changing climate (Tomiolo and Ward, 2018). Astronomical daylength itself is strictly latitude-dependent (**Figures 1A–D**), thus its interest in the context of global warming has been marginal at best. Atmospheric and meteorological factors, both impacted by rising temperatures, can locally affect crucial spectral light ratios for plant perception which implies an entanglement between light cues and climate change (Kotilainen et al., 2020).

The target of this study are arctic-alpine plant species (Reutimann et al., 2026). Given their peculiar distribution ranges and realized niches, arctic-alpine plant species offer a unique opportunity to investigate responses to climate change across different light environments. We aim to highlight the scarcity of experimental literature on population-specific responses of arctic-alpine plant species to photoperiod under the changing climate.

## 2 Arctic-alpine species: populations under the same bioclimate but across different ecological gradients

The need for shared terminology in arctic-alpine research is no new concept, nor is the arbitrariness of a series of terms, such as arctic, subarctic, and subalpine (Hustich, 1979). For instance, the Arctic Portal (https://arcticportal.org) reports seven different definitions for the ‘Arctic’. Similarly, not all authors accept the term ‘alpine tundra’ but prefer to differentiate between the tundra biome and the alpine biome (Testolin et al., 2020; Körner and Hiltbrunner, 2021). The concept of biome itself is a matter of debate (Mucina, 2019). This terminological arbitrariness influences the efficacy of keywords in literature research. We do not strive to solve the terminological issue nor to propose unequivocal definitions. We aim to bring to light a series of well documented ecological concepts relevant to arctic-alpine plant species, the habitats they inhabit and the abiotic similarities and differences they experience in their realized niches.

Billings and Mooney (1968) defined arctic-alpine plants as all species occurring beyond the treeline, both latitudinally and altitudinally. They share a common history shaped by the climatic oscillations of the Ice Age, which caused them to migrate at higher latitudes and altitudes during Interglacials and to either persist in glacial refuges or migrate to suitable habitats at lower altitudes and latitudes during Glaciations (Billings and Mooney, 1968; Reutimann et al., 2026). Arctic-alpine plant populations are currently scattered across the tundra biome, a bioclimatic region characterized by a cold bioclimate (i.e., the tundra bioclimate) which does not allow growth of trees and tall shrubs. Such conditions are realized both in polar regions and in the alpine altitudinal belt of mountain ranges across the world, with elevation of the alpine belt increasing from the polar circles towards the Equator (Loidi et al., 2022; Loidi, 2025; **Table 1**).

**Table 1:** Hierarchical bioclimatic classification regarding the tundra biome and its subbiomes, from Loidi, 2025 (DOI: 10.3897/VCS.139673).

Level 1		Level 2		Level 3	Thermotypes (Tp)						Ombrotypes (Io)						
Domain and macrobioclimate (Tp and Io)	Ecozone and bioclimate	Biomes	Subbiomes	Regional subbiomes	Infra	Thermo	Meso	Supra	Oro	Cryoro	Hyperarid (< 0.4)	Arid (0.4–1)	Subarid (1–2)	Dry (2–3.6)	Subhumid (3.6–6)	Humid (6–12)	Hyperhumid (> 12)
Cryocratic; Cold (Tp < 1000)	Polar and boreal ecozone; Tundral-boreal	1. Tundra	1a. Polar tundra	1a.1. Circumarctic polar tundra						X				X	X	X	X
				1a.2. Anctarctic polar tundra						X						X	X
			1b. Tundras of the temperate mountains in cryoro belt	1b.1. Eurasian mountains cryoro tundras						X						X	X
				1b.2. North American mountains cryoro tundras						X						X	X
				1b.3. Austral mountains cryoro tundras						X						X	X
			1c. Tundras of the tropical mountains in cryoro belt	1c. 1. Paleotropical mountains cryoro tundras						X						X	X
				1c. 2. Neotropical mountains cryoro tundras						X						X	X

All relevant definitions can be found in Loidi (2025). In brief, *Tp* is the sum of positive mean monthly temperatures; *thermotypes* are thermal regimes calculated from Tp; *Io* is the Rivas-Martínez Ombrothermic Index, based on the quotient of positive precipitation and Tp; *ombrotypes* are water-availability regimes calculated from Io; and *cryoro* is a thermotype characteristic of high altitudes and latitudes.

Despite the similar bioclimate, arctic-alpine plant populations can be found in several environmental gradients. For instance, temperature does not only change along latitudinal and altitudinal gradients, but also across micro-topographies (alongside water availability) depending on a few interconnected variables, such as slope, aspect, surface morphology, substrate type, wind exposure, snow cover, length of the snow-free period and incoming light (Leuschner and Ellenberg, 2018). Consequently, tundra habitats may exhibit a mosaic-like structure determined by different microenvironmental conditions which are in turn reflected in numerous plant communities, from snow-bed communities in concave terrains, to grasslands on flat or moderately inclined slopes, to communities of screes and wind-exposed ridges in peculiar habitats (Bliss, 1956; Leuschner and Ellenberg, 2018; Körner, 2023). As a consequence of such habitat heterogeneity, many arctic-alpine plant species exhibit relatively broad realized ecological niches. For example, *Saxifraga oppositifolia* L. can be found at alpine and subnival altitudes where it experiences shorter growing seasons due to low temperature (Larl and Wagner, 2006). *Salix reticulata* L., which usually inhabits snow-beds, can be also found in the drier habitats of alpine grasslands. *Bistorta vivipara* (L.) Delarbre can grow on substrates with very different geochemical profiles, from purely carbonatic to decalcified substrates and even on ophiolites (Cazzavillan et al., 2024).

Arctic-alpine plant populations can also be found across photoperiodic gradients. Photoperiod has been defined in different ways in the literature: (1) as a synonym of daylength; (2) as the ‘absolute photoperiod’, ranging from the first to the last photons the photoreceptors are able to perceive; (3) as the ‘photosynthetic photoperiod’, defined by sufficient radiation for the rate of photosynthesis to exceed the rate of respiration (Li and Gendron, 2025). We will use the second definition, so that photoperiod is both distinguishable from the astronomical concept of daylength and relevant to a wide array of plant processes besides photosynthesis. Because of the terrestrial tilt, arctic-alpine plant populations at different latitudes not only experience different daylengths but also different ranges of sun elevation during the day, which in turn affects the spectral quality of incident light (**Figure 1E**). Moreover, micro-topographical variables such as slope and aspect influence the amount of incident light (Bliss, 1956; Mooney and Billings, 1961; Keller and Körner, 2003; Leuschner and Ellenberg, 2018).

## 3 Light cues in a changing environment: a pivotal but understudied ecological factor

Among the ecological factors affecting plant responses to a changing environment, spectral light cues play a role in juvenile phenology (e.g., germination, de-etiolation, hypocotyl elongation), vegetative phenology (e.g., growth, leaf size and shape) and reproductive phenology (e.g., flowering). Spectral light also affects a wide array of developmental and physiological processes (e.g., vernalization and senescence, starch metabolism, chlorophyll content, stomatal density, chloroplast numbers) (Gendron and Staiger, 2023). Light cues exert regulatory effects on hormone production and on the circadian clock, allowing the assessment of nighttime by the plant (Gendron and Staiger, 2023; Li and Gendron, 2025). In addition, spectral ratios, such as R: FR (i.e., red/far-red), B: R (i.e., blue/red) and B: G (i.e., blue/green) have been suggested as a relevant yet underused class of measurements in applied photobiology, given their physiological implications for differential growth and circadian attuning (Parker et al., 2017; Kotilainen et al., 2020; Mølmann et al., 2021). This is especially relevant in high-latitude light environments. Circadian-compatible patterns have been observed for gas exchange and metabolic pools under continuous sunlight in the Low Arctic (68°N), whereas relaxation of circadian activity in pigment cycles has been observed in the High Arctic (78°N) (Patankar et al., 2013; Fernández-Marín et al., 2019). Although daylength did not change between those two studies, the light environment was indeed different and sun elevation differences did significantly alter the R: FR ratio the plants experienced during the Polar Day (**Figure 1F**). Divergent light responses and thermal stabilities of phytochrome B have been observed (Ikeda et al., 2021) between the arctic-alpine species *Cardamine bellidifolia* L. and the alpine species *Cardamine nipponica* Franch., exemplifying how not only the light environment but also the genetic and functional variability of the photoreceptors shape the plant response.

## 4 A critical review of extant studies on the photobiology of arctic-alpine plants

**Figure 2:**
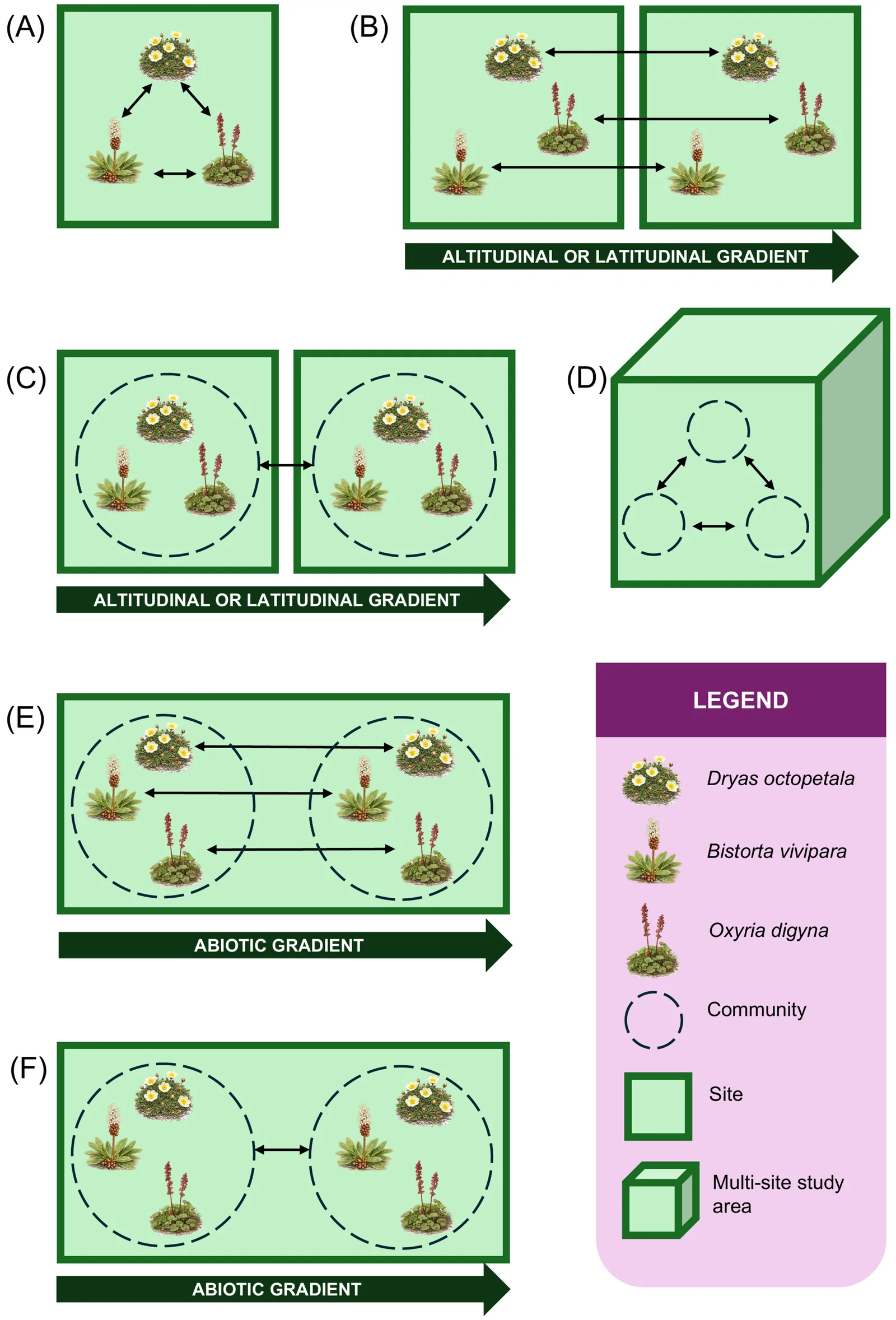
Models of comparison: **(A)** different species within the same site; **(B)** different populations in different sites along altitudinal or latitudinal gradients; **(C)** similar communities in different sites along altitudinal or latitudinal gradients; **(D)** similar communities within the tundra biome; **(E)** populations in the same site but along an abiotic gradient and in different communities, **(F)** different communities in the same site but along an abiotic gradient. Species icons are generated from field obtained photos and transformed with ChatGPT to remove background and emulate a botanical illustration.

The state of the art in photobiology relies mostly on the study of model organisms [e.g., *Arabidopsis thaliana* (L.) Heynh.] and crops (e.g., *Oryza sativa* L.) rather than of arctic-alpine plant species (Gendron and Staiger, 2023). We selected 22 papers concerning arctic-alpine species, which acknowledge, discuss, monitor or manipulate light (Mooney and Billings, 1961; Mooney and Johnson, 1965; Billings et al., 1971; Keller and Körner, 2003; Marchand et al., 2004; Heide, 2005; Larl and Wagner, 2006; Høye et al., 2007; Wagner and Simons, 2009; Cooper et al., 2011; Patankar et al., 2013; Abeli et al., 2015; Khorsand Rosa et al., 2015; Bjorkman et al., 2017; Parker et al., 2017; Fernández-Marín et al., 2019; Livensperger et al., 2019; Quaglia et al., 2020; Collins et al., 2021; Gehrmann et al., 2021; Crepaz et al., 2024; Brancaleoni et al., 2025). These studies offer a comprehensive overview of the wide range of approaches to the study of the response of arctic-alpine plant species to the changing environment (**Table 2**; **Appendix 1**: **Supplementary Table S5**). They showcase that light was rarely investigated to identify ecophenotypes in different light environments. Both single-site and multi-site study areas were chosen, some of the latter across latitudinal or altitudinal gradients (**Table 2**). In terms of phenotypic plasticity, single-site studies allow comparisons either among species or among populations and/or communities across abiotic gradients (**Figures 2A, E, F**; Keller and Körner, 2003; Høye et al., 2007; Cooper et al., 2011; Crepaz et al., 2024). Multi-site studies also allow comparisons across altitudinal and/or latitudinal gradients between different populations and/or communities (**Figures 2B–C**; Mooney and Billings, 1961; Mooney and Johnson, 1965; Billings et al., 1971; Heide, 2005; Wagner and Simons, 2009; Abeli et al., 2015; Brancaleoni et al., 2025) or between communities over the tundra biome (**Figure 2D**; Collins et al., 2021). Experimental designs included field-based (e.g., common gardens or monitoring sites) or controlled experiments (i.e., labs, greenhouses or research stations), with biotic and abiotic data being acquired both through field measurements and with remote-sensing techniques (e.g., phenocams; **Table 2**). Assessment of abiotic variables can be performed at macrohabitat, microhabitat or multi-scale level (**Table 2**). Within the same scale, abiotic and biotic variables can be further categorized and assessed with multiple direct and indirect measurements or with proxies (**Table 2**). Different definitions of the growing season were given, using either green-up or snowmelt for defining the start of the growing season and using either senescence or snowfall for defining the end of the growing season (Cooper et al., 2011; Crepaz et al., 2024) (Cooper et al., 2011; Crepaz et al., 2024). Such strong heterogeneity in the experimental approach not only hinders result comparison but also limits our understanding of the differences in phenotypic plasticity and adaptation among populations of arctic-alpine plant species.

**Table 2:** Visual table of arctic and/or alpine experimental contributions considering light.

Short citation	Locations	Design			Light		Temperature		Water		Snow	
Mooney and Billings, 1961	N–America, 15 S.L. ranging between 38–76°N	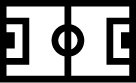	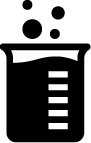		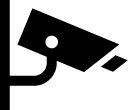	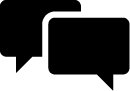		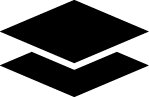	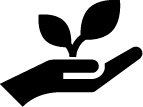		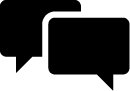	
Mooney and Johnson, 1965	California (37°N), Alaska (68°N)		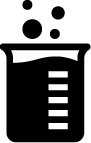		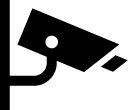	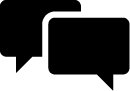		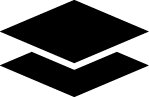				
Billings et al., 1971	N–America & Eur., 18 S.L. ranging between 35–81°N		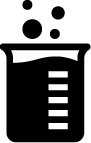		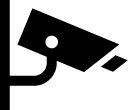	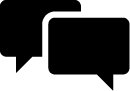		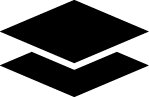	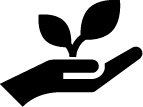			
Keller and Körner, 2003	Alps (47°N)		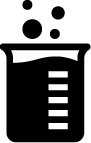		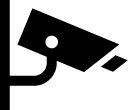	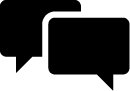		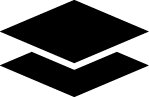			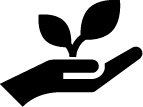	
Marchand et al., 2004	Greenland (74°N)	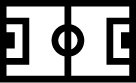				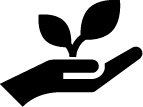		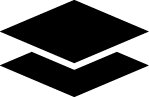	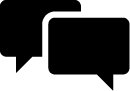	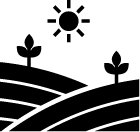		
Heide, 2005	4 sites ranging between 45–78°N		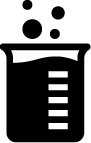		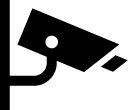	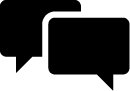		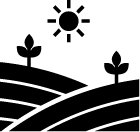	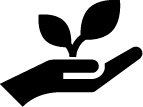			
Larl and Wagner, 2006	Alps (47°N)	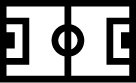				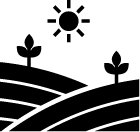		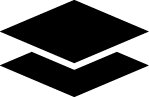		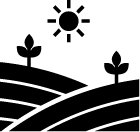		
Høye et al., 2007	Greenland (74°N)	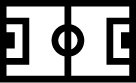				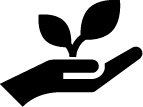		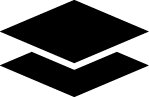	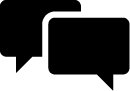			
Wagner and Simons, 2009	6 sites ranging between 40–78°N		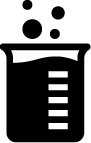		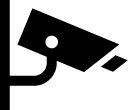	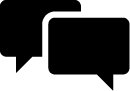		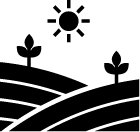	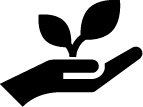			
Cooper et al., 2011	Svalbard (78°N)	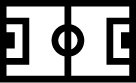	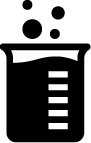		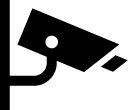	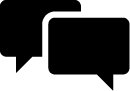		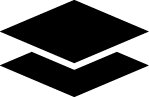			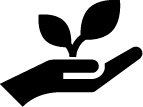	
Patankar et al., 2013	Alaska (68°N)	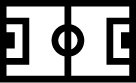			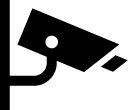	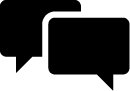		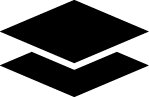				
Abeli et al., 2015	Europe, 4 S.L. ranging between 44–64°N		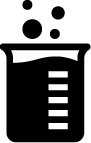		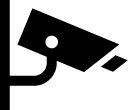	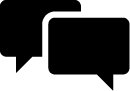		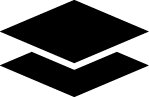	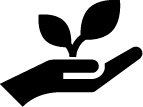			
Khorsand Rosa et al., 2015	Alaska (68°N)	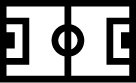				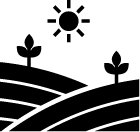		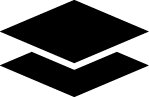			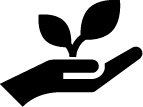	
Parker et al., 2017	Alaska, C.G. 68°N, S.L. ranging between 65– 70°N	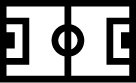				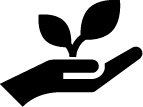		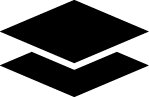				
Bjorkman et al., 2017	N–America & Eur, S.L. ranging between 68–79°N, C.G. 78°N	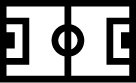	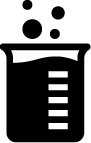		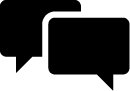	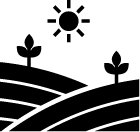		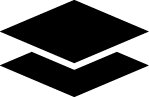				
Fernández-Marín et al., 2019	Svalbard (78°N)	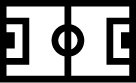			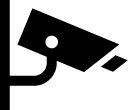	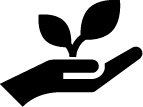		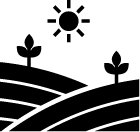		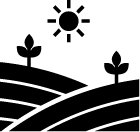		
Livensperger et al., 2019	Alaska (68°N)	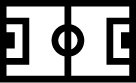				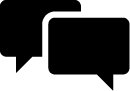		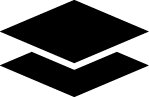			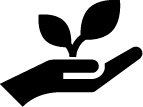	
Quaglia et al., 2020	Alps (45°N)	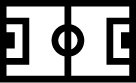				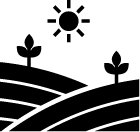		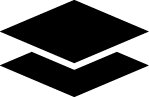				
Collins et al., 2021	18 sites ranging between 37–79°N	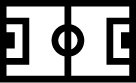			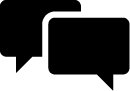	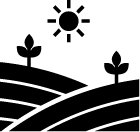		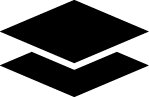				
Gehrmann et al., 2021	Svalbard (78°N)	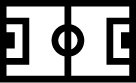				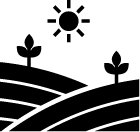		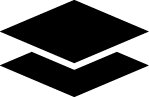			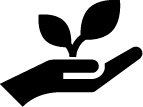	
Crepaz et al., 2024	Alps, 2 S.L. (46°N),	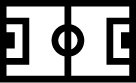				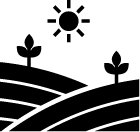		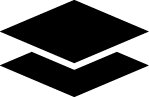				
Brancaleoni et al., 2025	Eur., 4 S.L. ranging between 44–64°N		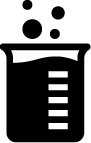		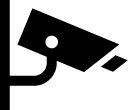	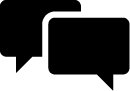		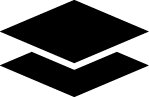	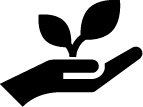			

Papers are ordered chronologically; their study area is recorded with the intent to underline the presence of either altitudinal or latitudinal gradients. A series of icons described in the panel’s legend graphically convey if the experimental designs are conducted in field or in controlled conditions, if remote sensing is used, if abiotic variables are discussed, recorded or manipulated and which scales are considered (i.e. macrohabitat, microhabitat, multi-scale). Common Garden (C.G.), Sampling Locations (S.L.), North America (N-America), Europe (Eur.). 
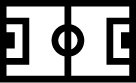
: in field experiment. 
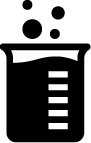
: laboratory/greenhouse-controlled experiment. 
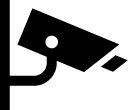
: remote-sensing techniques. 
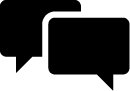
: acknowledged either in the introduction or in the discussion of the results. 

: record and monitor of environmental conditions. 
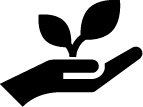
: manipulations (e.g. OTC, lamps, irrigation). 
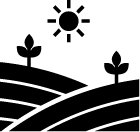
: macrohabitat scale (e.g. atmospheric air temperature, daylength, DOY, precipitation). 

: microhabitat scale (e.g. soil temperature, soil moisture, PAR, local snow-regime). 
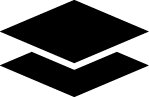
: multiple scales. Icons are included in the Microsoft Office package.

Most of the studies comparing arctic-alpine populations experiencing different photoperiodic regimes were carried out on *Oxyria digyna* (L.) Hill, which showed clear ecotypic differences across latitudinal gradients both in Europe and in North America (Mooney and Billings, 1961; Billings et al., 1971; Heide, 2005; Bjorkman et al., 2017). Coherently with the boundary of the Last Glacial Maximum (LGM), southern and northern phenotypes of *Oxyria digyna* (L.) Hill were found in North America, differing mainly by presence/absence of rhizomes, degree of inflorescence branching, leaf length-to-width ratio and stamen number (Mooney and Billings, 1961). Southern populations were reported (1) to be ‘strikingly more branched’ than the northern phenotypes, (2) to have a higher length-to-width leaf ratio, (3) to present flowers with only two stamens or a variable number of stamens compared to the northern populations presenting flowers with six stamens, and (4) to often lack rhizomes (Mooney and Billings, 1961). Similar differences were found in European populations of *Oxyria digyna*, for which different traits seemed to define ‘latitudinal boundaries’, possibly due to continental differences in the glacial history (Heide, 2005). For instance, the southernmost *Oxyria digyna* population in the Alps presented higher length-to-width ratio than the northern populations. However, inflorescence branch number decreased markedly only in the northernmost European population located in the Svalbard Islands (Heide, 2005). One of the major differences in morphotypes consisted in a variable number of stamens in the European High Arctic which was never observed in the North American High Arctic (Mooney and Billings, 1961; Heide, 2005). From a phenological standpoint, a clinal increase in photoperiodic requirements for flowering induction in parallel to a decrease in the number of flowers were reported for both European and North American *Oxyria digyna* populations (Mooney and Billings, 1961; Heide, 2005). The presence of rhizomes seemed to act as a compensation for lower sexual reproduction success at higher latitudes in favor of vegetative and clonal growth (Heide, 2005). Moreover, leaf-out and leaf senescence also followed a latitudinal cline with overall earlier onsets in high-latitude populations (Bjorkman et al., 2017). Metabolic acclimation to temperature has also been observed, with higher plasticity in alpine ecotypes (Mooney and Billings, 1961; Billings et al., 1971). Nonetheless, *Oxyria digyna* was believed to be well adapted to withstand negative effects of global warming (Heide, 2005). This meanwhile highlights the importance of adaptation to non-climatic factors in the context of climate change responses (Bjorkman et al., 2017). Ecotypic differences have also been observed across latitudinal gradients for *Eriophorum vaginatum* L (Parker et al., 2017), *Thalictrum alpinum* L (Mooney and Johnson, 1965). and *Viscaria alpina* (L.) G.Don (Abeli et al., 2015; Brancaleoni et al., 2025). The light environment can even change across altitudinal gradients. For instance, alpine and subnival populations of *Saxifraga oppositifolia* L. experience different snow-free season lengths. As a result, their reproductive phenophases are asynchronous, thus different populations are exposed to different daylengths and spectral regimes during the same phenophases (Larl and Wagner, 2006). Both *Eriophorum vaginatum* L. populations in Alaska and *Saxifraga oppositifolia* L. populations in the European Alps seemed to be adapted to the length of the growing season determined either by latitude or altitude, respectively (Larl and Wagner, 2006; Parker et al., 2017). Photoperiodic and spectral cues were indicated as possible regulating factors employed in phenological attuning to recurring seasonal patterns (Larl and Wagner, 2006; Parker et al., 2017). North American populations of *Thalictrum alpinum* L. showed different photoperiodic requirements, different photosynthetic light efficiency and temperature optima for photosynthesis, exemplifying the interplay of climatic and non-climatic environmental factors on plant physiology (Mooney and Johnson, 1965). Northern European populations of *Viscaria alpina* (L.) G. Don showed higher plasticity in their response to both drought and heat spells as a function of higher genetic variability, although clear local photoperiodic requirements have not been recorded among populations (Abeli et al., 2015; Brancaleoni et al., 2025). Lack of evidence for photoperiodic requirements may indicate a greater degree of phenotypic plasticity in northern European populations. Under global warming, phenotypic plasticity can buffer organisms against environmental change (Becklin et al., 2016). The degree of phenotypic plasticity of arctic-alpine plant populations is a good indicator of their ability to persist within a given photic regime, whereas adaptations to strict seasonal patterns may be detrimental under a changing climate due to thermal-photic mismatches (Huffeldt, 2020, Huffeldt, 2021). Such mismatches could not only affect their persistence within their current range, but can also reduce their capacity to colonize newly suitable habitats (Huffeldt, 2020). Phenological shifts in response to warming have been recorded in the Arctic under experimental conditions (Collins et al., 2021). Yet, the polar amplification predicted by the IPCC (Masson-Delmotte et al., 2021) could exacerbate thermal-photic mismatches for populations with a greater reliance on photoperiodism for seasonal entrainment of phenology at higher latitudes (Henry et al., 2022). So, greater attention to plant photobiology and photic constraints could substantially improve our understanding of arctic-alpine plant population responses to climate change.

## 5 Concluding remarks and future perspectives

The main conclusion of our review is that comparisons of populations rather than species (**Figures 2B, E**) are pivotal for investigating photobiologic differences and adaptations to the local light environments of arctic-alpine plant species. Our study also showed that experimental studies in this field are scarce to date. The limited pool of extant studies focused on population comparisons within a multi-site manipulative design, which is intrinsically challenging. Fieldwork in remote arctic or alpine areas implies a number of limitations including safety, logistics, local regulations, permit requirements (e.g., import and export of plant and soil material, weapon use, access to protected areas, etc.) and the respect of international agreements (e.g., the Nagoya Protocol). Therefore, reciprocal transplants, plant growth in common gardens and assisted migrations are not always feasible in arctic and alpine regions. We suggest consulting the INTERACT website (eu-interact.org) to tackle and disentangle the difficult organizational puzzle posed by multi-site arctic-alpine studies. As we experienced firsthand in the experimental phase of the project PHOTOPLANT (https://photoplant.unipv.it), field light manipulations pose additional challenges and limitations, which justify the common use of controlled manipulations (e.g., climate chambers) instead of field-based manipulations. The use of lamps to simulate longer daylengths requires reliable and stable power supply which can only approximate the everchanging spectral light environment, because lamps generally provide constant quality and intensity of the photosynthetically active radiation (Keller and Körner, 2003). However, neither field light manipulations nor multi-site experimental designs are an indispensable requirement for population comparisons. Transplants, if feasible, can indirectly manipulate photoperiod (Parker et al., 2017). This method can tackle night simulation, which is difficult to be artificially recreated in the Arctic. Manipulations of snow regime or altitudinal comparisons can also indirectly investigate photoperiodic requirements (Larl and Wagner, 2006; Bjorkman et al., 2017). Assessment of the light environment itself can be performed both indirectly (e.g., based on day of year) and directly [either by measuring spectral light or through physiological measurements related to photosynthesis (see **Appendix 1**: **Supplementary Table S5**)]. On the other hand, population-specific data from single-site studies can be compared in meta-analyses, provided that the experimental protocols are similar enough to allow reliable comparisons. In this regard, the Tundra Phenology Database (Prevéy et al., 2022) collected phenological data obtained using the ITEX protocol. Moreover, single-site species comparisons can also offer population-specific insights. For example, in the European Alps populations of *Saxifraga oppositifolia* L. proved to be insensitive to photoperiod, populations of *Oxyria digyna* (L.) Hill exhibited a long-day requirement and populations of *Sibbaldia procumbens* L. displayed a short-day requirement (Keller and Körner, 2003).

The scarcity of literature on photoperiodic requirements of arctic-alpine plant populations is not only contextual to the rising interest and concern in global warming, but also to: (1) disagreements on shared terminology; (2) evaluation of experimental protocols given the intrinsic interdisciplinarity and complexity of photobiological research; (3) critical assessment of logistical, technical and safety issues concerning fieldwork in remote areas. More population-level photobiological research is needed to predict future responses of arctic-alpine plant species to the changing climate, to increase the efficacy of assisted colonization, and to support crop science especially at the highest latitudes of the agroclimatic zone.
