# Supplementary material for: Shedding light on arctic-alpine plants: the understudied photobiological responses in the context of climate change
Source: Front Plant Sci. 2026 Aug 7;17:1920913. doi: 10.3389/fpls.2026.1920913 (PMC13494867; doi:10.3389/fpls.2026.1920913)
Supplement: Supplementary file 1 [file Table1.docx]

Supplementary Material

# 1 Supplementary Data for Figure 1

**Supplementary Table 1.** All data have been gathered from the United States Naval Observatory (<https://www.cnmoc.usff.navy.mil/usno/>). Arctic and alpine sites were chosen from the sites of the experimental papers cited in the review: Ny Ålesund, Svalbard, Norway (78.92°N, 11.92° E), Zackenberg, Greenland (74.46°N, –20.56°E), Toolik Lake, Alaska, USA (68.62°N, –149.58°E), Spåtind, Norway (61.08°N, 9.78°E), Mt. Sobretta (46.38°N, 10.42°E), Nam Co Station, Tibetan Plateau, China (30.76°N, 90.98°E) and Quito, Ecuador (–0.22°N, –78.51°E). Data is expressed as cumulative hours of the 21st day of each month of 2024, so that the conventional dates of the equinoxes and the solstices are mostly included while providing a monthly cadence to the measurements.

|  | **Ny Ålesund (79°N)** | **Zachenberg (74°N)** | **Toolik Lake (68°N)** | **Spåtind (61°N)** | **Mt. Sobretta (46°N)** | **Tibetan Plateau (30°N)** | **Quito (0°N)** |
| --- | --- | --- | --- | --- | --- | --- | --- |
| JAN | 0.00 | 0.00 | 3.78 | 6.85 | 9.20 | 10.47 | 12.13 |
| FEB | 1.93 | 6.87 | 8.58 | 9.60 | 10.65 | 11.27 | 12.12 |
| MAR | 11.57 | 12.67 | 12.55 | 12.35 | 12.23 | 12.17 | 12.11 |
| APR | 21.35 | 19.60 | 16.87 | 15.30 | 13.90 | 13.10 | 12.10 |
| MAY | 24.00 | 24.00 | 23.20 | 17.95 | 15.23 | 13.83 | 12.10 |
| JUN | 24.00 | 24.00 | 24.00 | 19.32 | 15.80 | 14.13 | 12.12 |
| JUL | 24.00 | 24.00 | 22.63 | 17.95 | 15.23 | 13.85 | 12.10 |
| AUG | 23.42 | 19.08 | 16.63 | 15.23 | 13.87 | 13.10 | 12.10 |
| SEP | 14.47 | 12.62 | 12.38 | 12.33 | 12.22 | 12.17 | 12.10 |
| OCT | 4.94 | 6.68 | 8.33 | 9.52 | 10.60 | 11.25 | 12.12 |
| NOV | 0.00 | 0.00 | 3.52 | 6.80 | 9.18 | 10.47 | 12.13 |
| DEC | 0.00 | 0.00 | 0.00 | 5.50 | 8.58 | 10.15 | 12.13 |

**Supplementary Table 2.** All data have been gathered from the United States Naval Observatory (<https://www.cnmoc.usff.navy.mil/usno/>). Arctic and alpine sites were chosen from the sites of the experimental papers cited in the review: Ny Ålesund, Svalbard, Norway (78.92°N, 11.92° E), Zackenberg, Greenland (74.46°N, –20.56°E), Toolik Lake, Alaska, USA (68.62°N, –149.58°E), Spåtind, Norway (61.08°N, 9.78°E), Mt. Sobretta (46.38°N, 10.42°E), Nam Co Station, Tibetan Plateau, China (30.76°N, 90.98°E) and Quito, Ecuador (–0.22°N, –78.51°E). Data is expressed as degrees of elevation at solar noon on the 21st of March, in order to show the maximum solar elevation at each latitude during the conventional spring equinox.

| **Solar hour** | **Ny Ålesund (79°N)** | **Zachenberg (74°N)** | **Toolik Lake (68°N)** | **Spåtind (61°N)** | **Mt. Sobretta (46°N)** | **Tibetan Plateau (30°N)** | **Quito (0°N)** |
| --- | --- | --- | --- | --- | --- | --- | --- |
| 00:00 | -11.5 |  |  |  |  |  |  |
| 01:00 | -11.3 |  |  |  |  |  |  |
| 02:00 | -10.5 |  |  |  |  |  |  |
| 03:00 | -8.9 |  |  |  |  |  |  |
| 04:00 | -6.7 | -8.9 | -10.4 |  |  |  |  |
| 05:00 | -4.1 | -5.4 | -5.3 | -10.1 |  |  |  |
| 06:00 | -1.3 | -1.5 | 0.6 | -3 | -4.1 | 0 | -5.4 |
| 07:00 | 1.9 | 2.8 | 5.7 | 4.4 | 6.4 | 12.4 | 9.7 |
| 08:00 | 4.5 | 6.5 | 10.8 | 11.4 | 16.5 | 25 | 24.6 |
| 09:00 | 6.9 | 10 | 15.3 | 17.8 | 25.9 | 37.1 | 39.6 |
| 10:00 | 8.9 | 12.9 | 18.9 | 23.2 | 34.2 | 47.9 | 54.6 |
| 11:00 | 10.2 | 14.9 | 21.2 | 27.2 | 40.5 | 56.2 | 69.6 |
| 12:00 | 10.8 | 16 | 22.1 | 29.2 | 43.8 | 59.7 | 84.6 |
| 13:00 | 10.7 | 16 | 21.5 | 29.2 | 43.6 | 56.8 | 80.4 |
| 14:00 | 9.9 | 14.9 | 19.4 | 27 | 39.7 | 49 | 65.4 |
| 15:00 | 8.3 | 12.9 | 16.1 | 23 | 33.1 | 38.3 | 50.4 |
| 16:00 | 6.3 | 10 | 11.7 | 17.5 | 24.6 | 26.4 | 35.4 |
| 17:00 | 3.8 | 6.6 | 6.7 | 11 | 15 | 13.8 | 20.4 |
| 18:00 | 1.2 | 2.8 | 1.6 | 4.1 | 5 | 1.3 | 5.5 |
| 19:00 | -2.1 | -1.4 | -4.2 | -3.3 | -5.5 | -11.9 | -9.6 |
| 20:00 | -4.8 |  | -9.3 | -10.3 |  |  |  |
| 21:00 | -7.2 |  |  |  |  |  |  |
| 22:00 | -9.2 |  |  |  |  |  |  |
| 23:00 | -10.5 |  |  |  |  |  |  |
| 00:00 |  |  |  |  |  |  |  |

**Supplementary Table 3.** All data have been gathered from the United States Naval Observatory (<https://www.cnmoc.usff.navy.mil/usno/>). Arctic and alpine sites were chosen from the sites of the experimental papers cited in the review: Ny Ålesund, Svalbard, Norway (78.92°N, 11.92° E), Zackenberg, Greenland (74.46°N, –20.56°E), Toolik Lake, Alaska, USA (68.62°N, –149.58°E), Spåtind, Norway (61.08°N, 9.78°E), Mt. Sobretta (46.38°N, 10.42°E), Nam Co Station, Tibetan Plateau, China (30.76°N, 90.98°E) and Quito, Ecuador (–0.22°N, –78.51°E). Data is expressed as degrees of elevation at solar noon on the 21st of June, in order to show the maximum solar elevation at each latitude during the conventional summer solstice.

| **solar hour** | **Ny Ålesund (79°N)** | **Zachenberg (74°N)** | **Toolik Lake (68°N)** | **Spåtind (61°N)** | **Mt. Sobretta (46°N)** | **Tibetan Plateau (30°N)** | **Quito (0°N)** |
| --- | --- | --- | --- | --- | --- | --- | --- |
| 00:00 | 12.50 | 8.1 | 2.3 | -5.4 |  |  |  |
| 01:00 | 12.60 | 8.2 | 2.9 | -5.1 |  |  |  |
| 02:00 | 13.50 | 9.2 | 4.8 | -3.2 |  |  |  |
| 03:00 | 15.00 | 11.2 | 7.8 | 0.7 | -11.4 |  |  |
| 04:00 | 17.10 | 13.9 | 11.8 | 5.3 | -4.3 | -10.6 |  |
| 05:00 | 19.60 | 17.2 | 16.5 | 11.1 | 4.3 | 0.8 |  |
| 06:00 | 22.40 | 21 | 21.8 | 17.8 | 13.5 | 12.2 | -3.7 |
| 07:00 | 25.20 | 25 | 27.2 | 24.9 | 23.4 | 24.5 | 10.1 |
| 08:00 | 28.00 | 28.9 | 32.5 | 32.1 | 33.7 | 37.2 | 23.7 |
| 09:00 | 30.50 | 32.5 | 37.4 | 39 | 44 | 50 | 37 |
| 10:00 | 32.50 | 35.6 | 41.3 | 45.1 | 53.7 | 62.8 | 49.4 |
| 11:00 | 33.90 | 37.8 | 43.9 | 49.8 | 62 | 75.2 | 60 |
| 12:00 | 34.50 | 38.9 | 44.8 | 52.2 | 66.7 | 82.7 | 66 |
| 13:00 | 34.30 | 38.8 | 43.9 | 51.8 | 65.7 | 74.4 | 64 |
| 14:00 | 33.30 | 37.4 | 41.3 | 48.8 | 59.5 | 62 | 55.4 |
| 15:00 | 31.50 | 35.1 | 37.4 | 43.8 | 50.7 | 49.1 | 43.7 |
| 16:00 | 29.20 | 31.9 | 32.6 | 37.4 | 40.7 | 36.3 | 30.8 |
| 17:00 | 26.50 | 28.2 | 27.3 | 30.4 | 30.4 | 23.7 | 17.3 |
| 18:00 | 23.70 | 24.2 | 21.8 | 23.1 | 20.2 | 11.4 | 3.8 |
| 19:00 | 20.90 | 20.2 | 16.6 | 16.1 | 10.4 | 0.1 | -10.2 |
| 20:00 | 18.20 | 16.5 | 11.8 | 9.6 | 1.6 | -11.3 |  |
| 21:00 | 15.90 | 13.3 | 7.8 | 4 | -6.8 |  |  |
| 22:00 | 14.20 | 10.7 | 4.8 | -0.1 |  |  |  |
| 23:00 | 13.00 | 9 | 3 | -3.8 |  |  |  |
| 00:00 | 12.50 | 8.1 | 2.3 |  |  |  |  |

**Supplementary Table 4.** All data have been gathered from the United States Naval Observatory (<https://www.cnmoc.usff.navy.mil/usno/>). Arctic and alpine sites were chosen from the sites of the experimental papers cited in the review: Ny Ålesund, Svalbard, Norway (78.92°N, 11.92° E), Zackenberg, Greenland (74.46°N, –20.56°E), Toolik Lake, Alaska, USA (68.62°N, –149.58°E), Spåtind, Norway (61.08°N, 9.78°E), Mt. Sobretta (46.38°N, 10.42°E), Nam Co Station, Tibetan Plateau, China (30.76°N, 90.98°E) and Quito, Ecuador (–0.22°N, –78.51°E). Data is expressed as degrees of elevation at solar noon on the 21st of December, in order to show the maximum solar elevation at each latitude during the conventional winter solstice.

| **solar hour** | **Ny Ålesund (79°N)** | **Zachenberg (74°N)** | **Toolik Lake (68°N)** | **Spåtind (61°N)** | **Mt. Sobretta (46°N)** | **Tibetan Plateau (30°N)** | **Quito (0°N)** |
| --- | --- | --- | --- | --- | --- | --- | --- |
| 00:00 |  |  |  |  |  |  |  |
| 01:00 |  |  |  |  |  |  |  |
| 02:00 |  |  |  |  |  |  |  |
| 03:00 |  |  |  |  |  |  |  |
| 04:00 |  |  |  |  |  |  |  |
| 05:00 |  |  |  |  |  | -9.8 |  |
| 06:00 |  |  |  |  |  | -10.6 | -2.7 |
| 07:00 |  |  |  |  | -9.7 | 1.5 | 11.1 |
| 08:00 |  |  | -11.5 | -9.2 | -0.1 | 12.1 | 24.7 |
| 09:00 |  |  | -7.5 | -3.5 | 7.4 | 21.6 | 37.9 |
| 10:00 |  | -10.5 | -4.5 | 1.3 | 13.7 | 29.3 | 50.4 |
| 11:00 |  | -8.8 | -2.6 | 4.2 | 18.1 | 34.3 | 60.9 |
| 12:00 |  | -8 | -2.1 | 5.5 | 20.1 | 35.8 | 66.6 |
| 13:00 |  | -8.1 | -2.8 | 5.2 | 19.5 | 33.6 | 64.1 |
| 14:00 |  | -9.2 | -4.8 | 3.3 | 16.4 | 28 | 55.1 |
| 15:00 |  | -11.2 | -7.9 | 0 | 11.1 | 19.9 | 43.2 |
| 16:00 |  |  | -12 | -5.4 | 4.1 | 10.1 | 30.2 |
| 17:00 |  |  |  | -11.4 | -4.6 | -1.1 | 16.7 |
| 18:00 |  |  |  |  | -11.7 |  | 3.2 |
| 19:00 |  |  |  |  |  |  | -10.8 |
| 20:00 |  |  |  |  |  |  |  |
| 21:00 |  |  |  |  |  |  |  |
| 22:00 |  |  |  |  |  |  |  |
| 23:00 |  |  |  |  |  |  |  |
| 00:00 |  |  |  |  |  |  |  |

# 2 Supplementary Data for Table 2

**Supplementary Table 5.** Disambiguation of variable categories and methods of assessment. Both abiotic and biotic variables are assessed using a variety of methods in contributions referenced in Table 2. (a) Parameters concerning light and light-dependent physiology. (b) Parameters regarding temperature. (c) Parameters regarding water. (d) Parameters regarding snow. (e) Parameters regarding plant phenology and morphology at different scales. DOY: Day Of the Year. PAR: Photosynthetically Active Radiation. WUE: Water Use Efficiency. PFD: Photon Flux Density. PPFD: Photosynthetic Photon Flux Density. PSII: photosystem II. F_v_/F_m_: maximum PSII efficiency. Φ_PSII_: operating efficiency of PSII. A_max_: maximum photosynthetic capacity at saturating light. R_d_: dark respiration). g_s_: stomatal conductance. Chl: Chlorophyll. T_max_: maximum temperature. GDD: Growing Degrees Days. TDD: Thawing Degree Days. CDD: Cold Degree Days. OTC: Open Top Chambers. FATI: Free Air Temperature Increase. VWC: Volumetric Water Content. VSWC: Volumetric Soil Water Content. VPD: Water Pressure Deficit. RWC: Relative Water Content. DFSM: Days From SnowMelt. DOSM: Day Of SnowMelt. DSM: Days Since Snowmelt. WGR: Weekly Growth Rate. RSR: Root/Shoot Ratio. TGC: Total Green Cover. HPGC: Higher Plant Green Cover. SLA: Specific Leaf Area. TMF: Transition to Main Flowering. GSL: Growing Season Length. SOS: Start Of Season. SPS: Start of Peak of Season. POS: Peak Of Season. EPS: End of the Peak of the Season. EOS: End Of Season. PDR: Phenological Development Rate. P_iv_: Phenotypic plasticity index. GCC: Green Chromatic Coordinate. NDVI: Normalized Difference Vegetation Index.

|  | **CATEGORY** | **Variables** |
| --- | --- | --- |
| **(a)** | Indirect | DOY, microtopography |
|  | Treatments | Lamps (fixed daylength, fixed PAR, fixed intensity), shading (direct with cloths, indirect from rain shelters) |
|  | Spectral light | PAR, SWR |
|  | Photosynthesis and respiration | Net photosynthesis (μmol CO_2_ m^-2^ s^-1^), WUE (μmol CO_2_ mol^-1^ H2O) PFD, PPFD, F_v_/F_m_, Φ_PSII_, A_max_, R_d_, g_s_, mitochondrial oxidation rate |
|  | Light intensity | Incident light & indirect effects of other treatments (e.g. raining shelters, shading treatments) |
|  | Light reflectance | Leaf reflectance |
|  | Metabolites | Carbohydrates (disaccharides and polysaccharides), lipid-derived (e.g. Tocopherol), pigments (Chl a, Chl b, carotenoids, xantophylles) |
|  | Derived | Chl ratios, xanthophylles ratios, other pigment ratios, net carbon balance, degree of acclimation, compensation point, T_max_ of photosynthesis, vigor index |
| **(b)** | Microenvironmental | Near-ground, surface, below-ground, leaf surface or vegetation surface |
|  | Macroenvironmental | Above-ground (≥ 30 cm) |
|  | Derived | Thermal sums (GDD, TDD, CDD, means (coldest quarter, phenophase related, seasonal) |
|  | Treatments | OTC, FATI, heating wires, green-house, controlled chambers (growth, overwintering), elevation gradients |
| **(c)** | Microenvironmental | VWC, VSWC, leaf water content, soil moisture, gravimetric soil water content, stomatal conductance |
|  | Macroenvironmental | Precipitation, relative humidity |
|  | Treatments | Watering, rain shelters, wet filter paper |
|  | Defining habitat characteristic | Moist acidic tussock tundra, mesic heaths, dry heaths |
|  | Derived | Water balance, wetness index, desiccation trajectory, average daytime VPD, RWC |
| **(d)** | DOY related | DFSM, DOSM, DSM, GSL, snow–cover duration, snow regime |
|  | Treatments | Physical removal, tents, fences, black shade cloth, overwintering chambers |
| **(e)** | Counts | Leaves, flowers, seeds per flower, stamen, mesophyll cells |
|  | Presence/absence | Inflorescence branching, rhizomes presence, active leaf buds’ presence, |
|  | Morphometric measurements | Leaf, stems, stamens, carpels, petioles, flower buds |
|  | Phenophases | Early stages of phenology (germination, seedling growth), vegetative phenology (greening s.l., peak growth, senescence), reproductive phenology (flowering, fruiting, seed maturity and dispersal) |
|  | DOY/DFSM related | Onsets, peaks, endings, durations (prefloration interval), rates, synchronies/asynchronies |
|  | Weights | Biomasses (dry weights, leaf weights, seed weights) |
|  | Developmental | Root-tip mitoses, pollen mother cell meioses, flower bud stage (prefloration stages), vital buds, leaf emergence, fruit development, |
|  | Target tissues and structures | Root, root meristem, leaf buds, floral buds, floral apices, fruits, seeds, stoma |
|  | Tissutal parameters | Mesophyll cell number, palisade cell length, stomatal area, stomatal length, cuticular characteristics, root-tip mitoses |
|  | Derived | Phenotypic plasticity index, proportions, mortality/vitality, probabilities, WGR, RSR, TGC, HPGC, SLA, TMF, GSL, SOS, SPS, POS, EPS, EOS, PDR, P_iv_ |
|  | Community | Cover (%), hue-shift, GCC index, NDVI |
